# Ag@ZnO Nanoparticles Induce Antimicrobial Peptides and Promote Migration and Antibacterial Activity of Keratinocytes

**DOI:** 10.1021/acsinfecdis.0c00903

**Published:** 2021-03-29

**Authors:** Rakesh Kumar Majhi, Soumitra Mohanty, Md. Imran Khan, Amrita Mishra, Annelie Brauner

**Affiliations:** † Department of Microbiology, Tumor and Cell Biology, Karolinska Institutet, 171 77 Stockholm, Sweden; ‡ Division of Clinical Microbiology, Karolinska University Hospital, 171 76 Stockholm, Sweden; § School of Biotechnology, Kalinga Institute of Industrial Technology (KIIT), Bhubaneswar, 751024 Odisha, India

**Keywords:** silver nanoparticle, hBD2, RNase7, wound healing, infection, innate immunity

## Abstract

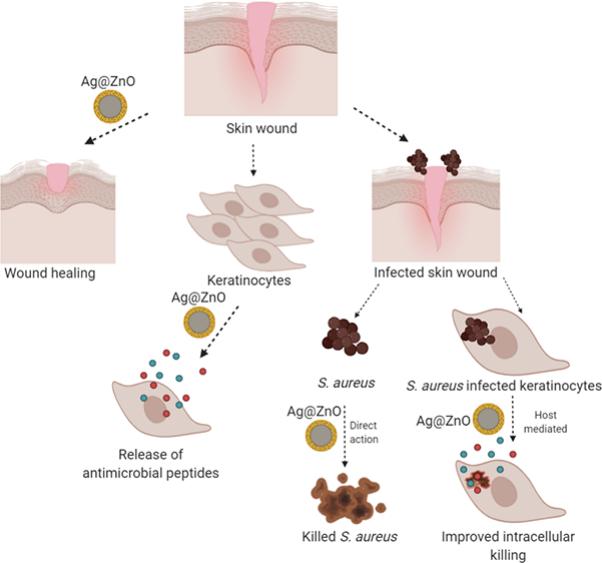

Antibacterial activity of silver nanoparticles is often associated with toxicity to the host. We here report that noncytotoxic doses of silver nanoparticles coated with zinc oxide, Ag@ZnO, can stimulate proliferation and migration of human keratinocytes, HaCaT, with increased expression of Ki67 and vinculin at the leading edge of wounds. Interestingly, Ag@ZnO stimulates keratinocytes to produce the antimicrobial peptides hBD2 and RNase7, promoting antibacterial activity against both extracellular and intracellular *Staphylococcus aureus* isolated from wounds. Overall, these results suggest that Ag@ZnO has the potential to significantly improve treatment outcomes in clearing wound infection.

The natural barrier formed by keratinocytes of the skin can be compromised by wounds. To promote wound healing, active proliferation and migration of keratinocytes is necessary but can be impaired by factors like underlying diseases, ongoing medications, aging, and bacterial infection.^1^*Staphylococcus aureus* colonizes the skin but often causes infection and is the most common bacterial infection that impedes wound healing in compromised skin. Although this does not always require treatment, increasing antibiotic resistance is alarming and calls for new treatment strategies. Metallic nanoparticles are emerging as potential alternatives,^2^ supported by their beneficial effects in treating infections in rodent wound models.^3^ However, the cytotoxicity of Ag^+^ ions released from silver nanoparticles toward human cells has hindered their clinical application, necessitating development of biocompatible silver nanocomposites.^4^

Previously, we synthesized silver nanoparticles coated with a thin layer of zinc oxide (Ag@ZnO) with an average size range of 42 nm, which demonstrated antifungal activity and reduced toxicity toward human epidermal cells.^5^ However, to be suitable for dermatological application on ulcers, it is essential to evaluate the effect of Ag@ZnO on viability of keratinocytes, possibility of promoting wound healing and efficacy in clearing infections by *S*. *aureus* strains often found in wounds.

In the current study, we demonstrated that Ag@ZnO can promote wound healing by increasing proliferation and migration of keratinocytes. Our study demonstrates for the first time that Ag@ZnO can trigger the host antimicrobial peptides human beta defensin-2 (hBD2) and RNase7, thereby improving intracellular lysosomal degradation and direct extracellular bactericidal effect on clinical *S*. *aureus* strains isolated from skin wounds.

Interestingly, human keratinocyte cell line HaCaT treated with 100 μg/mL Ag@ZnO for 24 h promoted keratinocyte proliferation by 25% with unaltered cellular morphology (Figure 1A, B) and increased cell migration to the wound area (Figure 1C), resulting in significant *in vitro* wound closure (Figure 1D). The enhanced migration was mirrored by increased expression of the keratinocyte proliferation marker Ki67 (Figure 1E) and the cell migration promoting protein vinculin (Figure 1F) at the leading edges of the wound within 24 h of Ag@ZnO treatment.^6−8^ Higher concentrations resulted in significant reduction of cell viability, disruption of actin cytoskeleton, and genotoxicity reflected by nuclear fragmentation (Figure 1A, B).

**Figure 1:**
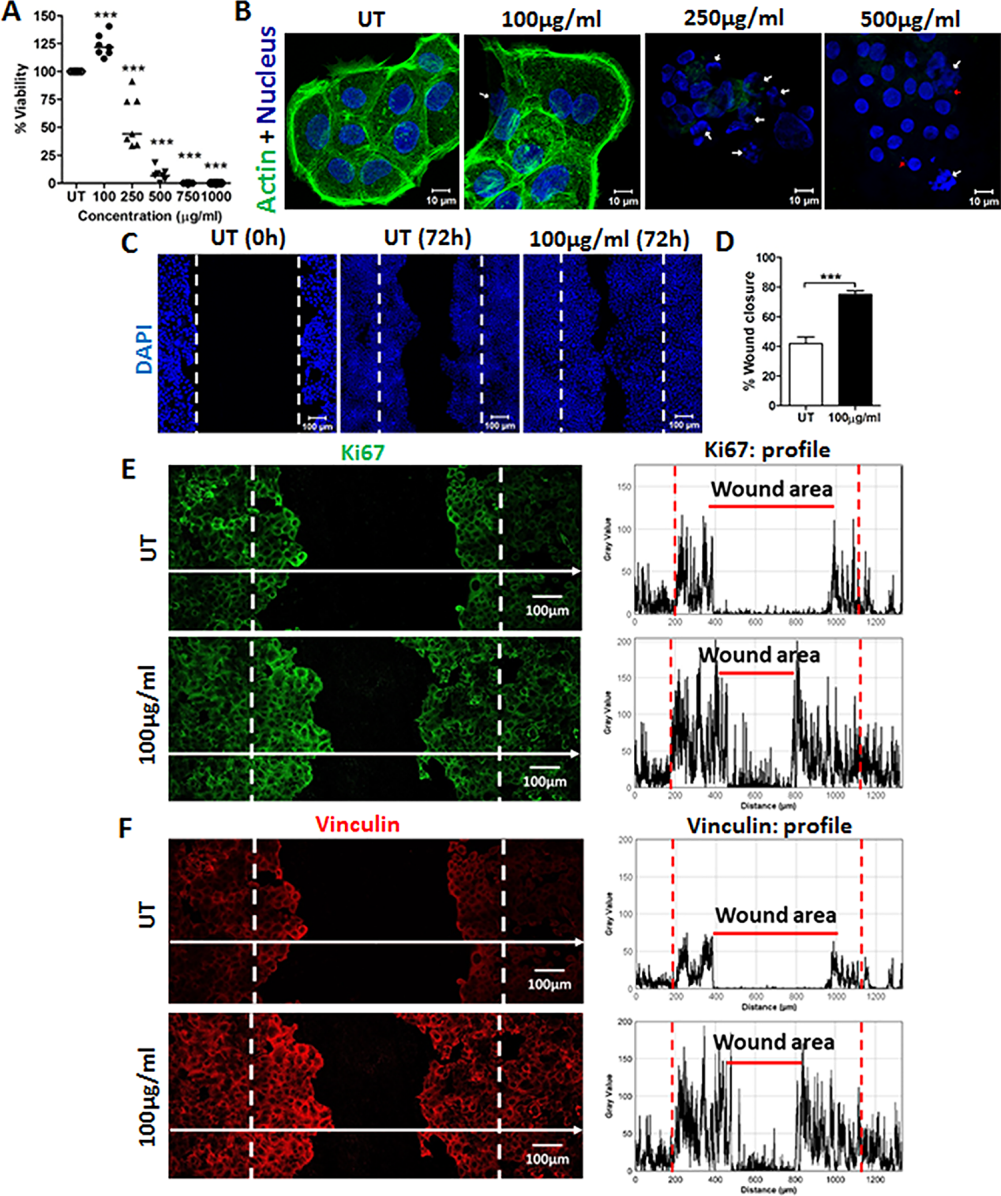
Ag@ZnO nanoparticles promote wound healing *in vitro*. (A) XTT assay depicts percentage of viable HaCaT cells treated with increasing concentration of Ag@ZnO for 24 h (*n* = 3, ****p < 0.0001) (B) Representative confocal images of HaCaT cells treated with Ag@ZnO for 24 h, filamentous actin (green), nucleus (blue). White arrows depict genotoxicity by nuclear fragmentation. Red arrows depict micronuclei formation (*n* = 3). (C) Representative confocal images of HaCaT cells migrating into the wound area at 72 h, nucleus (blue). (D) Percentage of wound closure compared to initial (0 h) wound area (*n* = 3, *p* < 0.0001, unpaired *t* test). Representative images of Ki67 (green) (E) and vinculin (red) (F) expression at 24 h in untreated and 100 μg/mL Ag@ZnO treated wounds. White dotted lines show the initial wound area. Corresponding expression profile plots drawn from the region depict higher Ki67 and vinculin expression at the leading edges (*n* = 2).

Healthy, intact skin is capable of preventing bacterial infections partly by secreting antimicrobial peptides.^9^ Besides having antimicrobial activity, they also promote wound healing and strengthen the epithelial barrier function.^10,11^ We therefore investigated if Ag@ZnO induced the antimicrobial peptides hBD2, RNase7, and psoriasin, widely expressed in keratinocytes.^10^ Ag@ZnO treatment increased hBD2 (*DEFB4A*) and RNase7 (*RNASE7*) mRNA and protein levels compared to untreated control (Figure 2A–F). Notably, this is the first report demonstrating that silver nanoparticles can increase expression of both hBD-2 and RNase7, which implies that Ag@ZnO can also activate the skin innate immunity against bacterial infection. Silver nanoparticles induce psoriasin expression in epidermal cells^12^ but not in keratinocytes (data not shown), likely due to differences in chemical composition and cells used.

**Figure 2:**
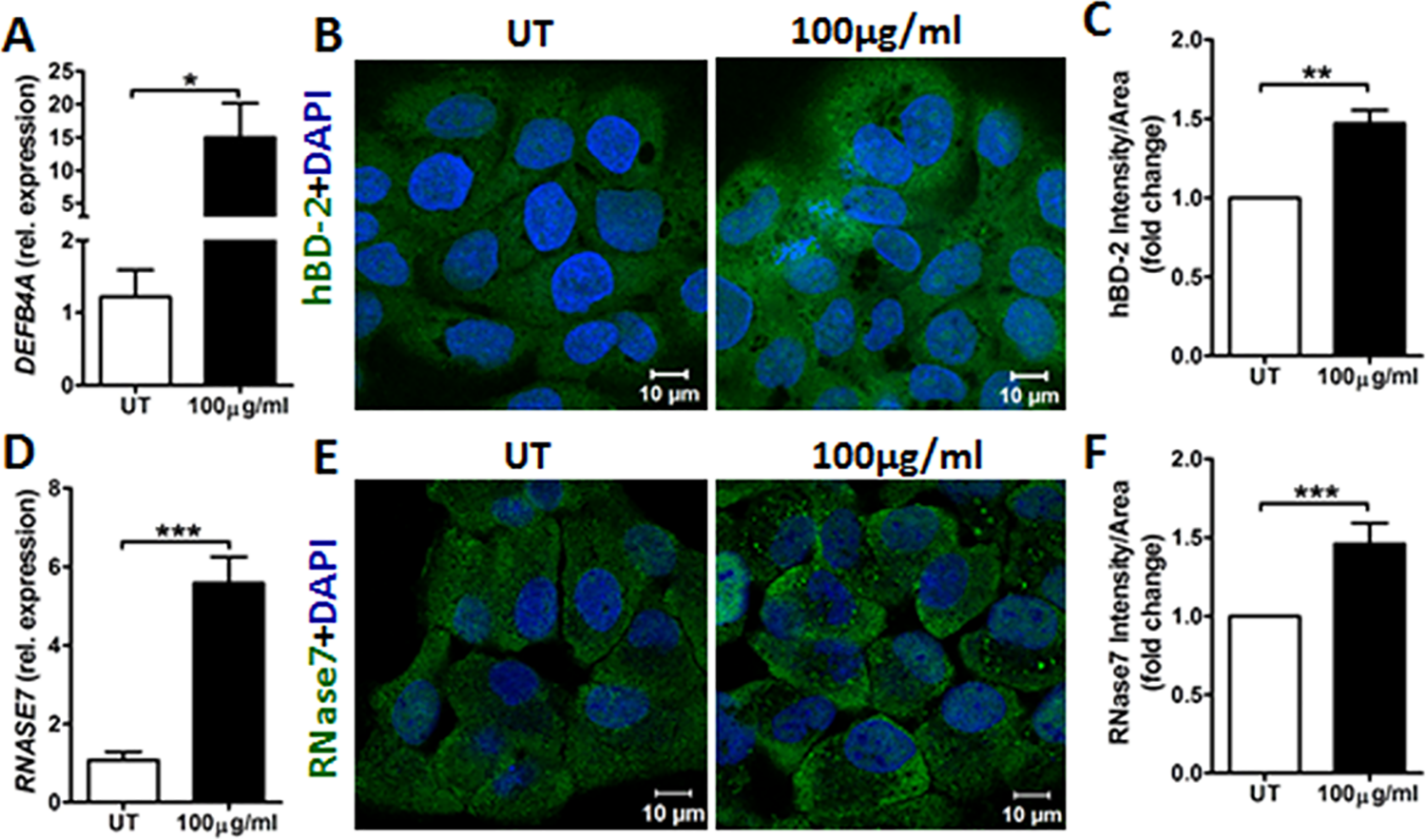
Ag@ZnO stimulates production of antimicrobial peptides hBD2 and RNase7 in keratinocytes. (A) *DEFB4A* expression shown as fold change in untreated and Ag@ZnO treated HaCaT by qPCR (*n* = 3, *p < 0.05). (B) Representative confocal images of hBD2 expression (green) in HaCaT cells counterstained with DAPI (blue). (C) Intensity quantification of hBD2 expression from 9 random view fields, shown as intensity per unit area (*n* = 3, **p < 0.01). (D) *RNASE7* expression shown as fold change in HaCaT cells by qPCR (*n* = 3, ***p < 0.001). (E) Representative confocal images of RNase7 expression (green) in HaCaT cells counterstained with DAPI (blue). (F) Intensity quantification of RNase7 expression from 9 random view fields, shown as intensity per unit area (*n* = 3, ***p < 0.001).

Silver nanoparticles show direct antibacterial activity by damaging the bacterial cell membrane, increasing reactive oxygen species, and also disrupting the bacterial cytoskeleton.^3,13^*S*. *aureus* is one of the most common microorganisms complicating skin wound healing. Therefore, we investigated the direct antibacterial effect of Ag@ZnO on 50 clinical *S*. *aureus* strains and the ATCC type strain 29213, all isolated from wounds. We observed significant reduction in *S*. *aureus* viability upon Ag@ZnO treatment for 24 h (Figure 3A) but not complete bacterial clearance. The clinical isolates and *S*. *aureus* type strain showed similar responses to direct action of Ag@ZnO. Hence, using only the type strain, we investigated whether better antibacterial activity can be achieved by keratinocytes stimulated with Ag@ZnO. Significant reduction in viability of *S*. *aureus* was observed upon incubation with conditioned media from Ag@ZnO treated keratinocytes (Figure 3B). Because *S*. *aureus* can also invade keratinocytes and survive intracellularly,^14^ we investigated the possible intracellular effect of Ag@ZnO. Interestingly, treatment with Ag@ZnO significantly reduced the survival of *S*. *aureus* in infected keratinocytes (Figure 3C). It is known that *S*. *aureus* is susceptible to hBD2 *in vitro*,^15^ while RNase7 produced by keratinocytes prevents skin colonization by *S*. *aureus*.^16^ Both hBD2 and RNase7 are active intracellularly and can be secreted by the keratinocytes. Therefore, Ag@ZnO induced hBD2 and RNase7 could contribute to its extracellular and intracellular antibacterial activity. It is worth noting that *S*. *aureus* tends to evade degradation in lysosomal compartments of keratinocytes.^17^ However, increased colocalization of fluorescently labeled *S*. *aureus* with the lysosomes was observed within 1.5 h of Ag@ZnO treatment, indicating activation of the endocytic pathway and increased lysosomal degradation of the intracellular bacteria (Figure 3D, E).

**Figure 3:**
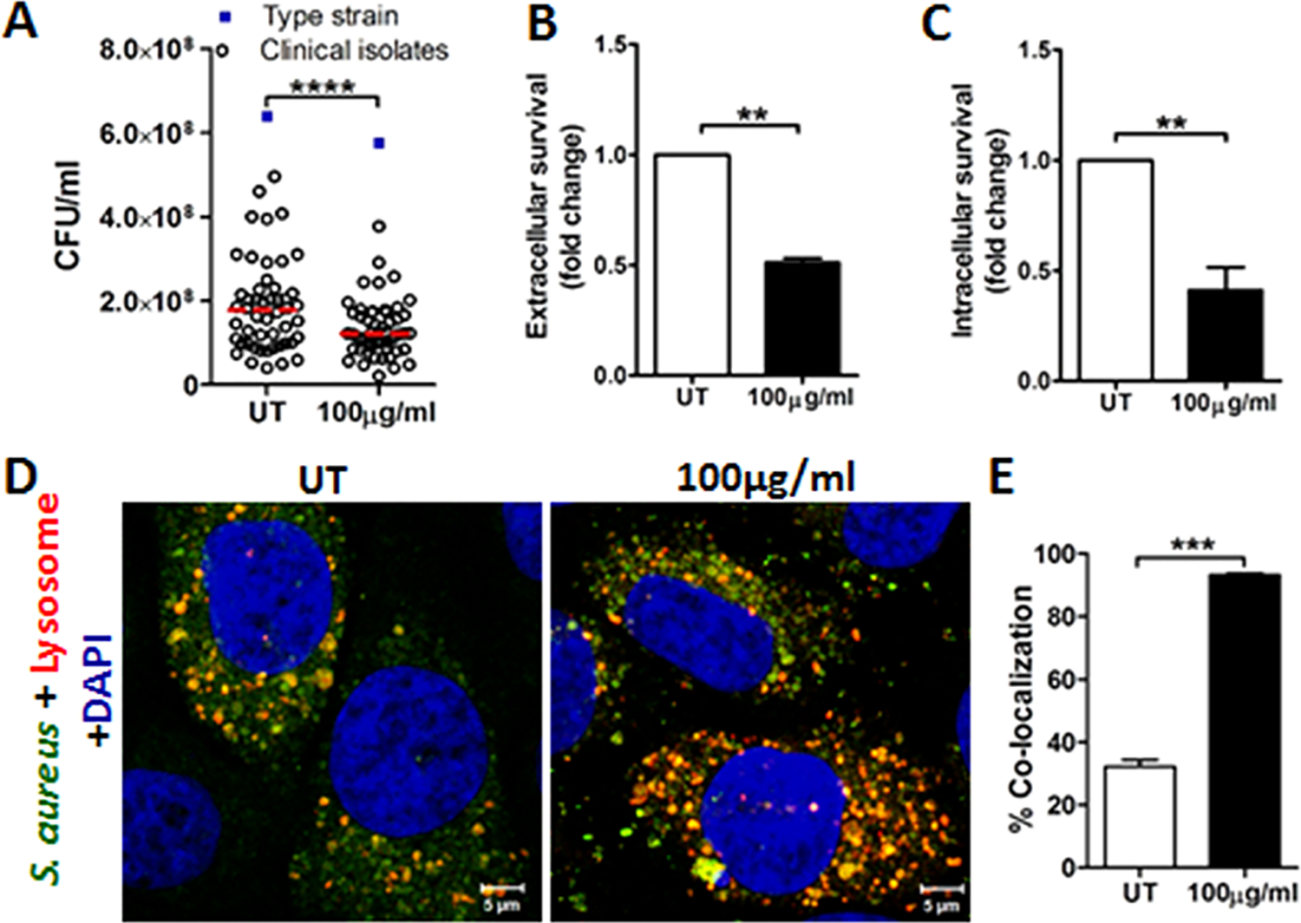
Ag@ZnO promotes antibacterial activity of keratinocytes. (A) Viable count (CFU/mL) of 50 clinical *S*. *aureus* isolates (open black circles) and ATCC 29213 type strain (blue squares) from wounds in untreated (UT) and 100 μg/mL of Ag@ZnO treated bacteria at 24 h (*n* = 51, ****p < 0.00001, paired *t* test). (B) Survival of *S*. *aureus* in conditioned media from HaCaT cells with and without Ag@ZnO treatment for 24 h (*n* = 3, **p < 0.01). (C) Intracellular survival of *S*. *aureus* in HaCaT cells with and without Ag@ZnO treatment for 24 h (*n* = 4, **p < 0.01). (D) Representative confocal images of HaCaT cells showing colocalization of *S*. *aureus* (green) and lysosomes (Red). (E) Percentage of *S*. *aureus* colocalizing with lysosomes (11 random view-fields, *n* = 2, ***p < 0.001, unpaired *t* test).

We here demonstrate that nontoxic concentrations of Ag@ZnO can support the skin by increasing the expression of antimicrobial peptides hBD2 and RNase7 and lysosomal degradation of intracellular bacteria and promoting wound closure. Rational designing of similar silver nanoparticles can be candidates for clinical use in skin therapy.

## Methods

### Cell Culture and Viability Assay

HaCaT cells were cultured in DMEM with 5% fetal bovine serum (Life Technologies) at 37 °C, 5% CO_2_. Lyophilized nanoparticles were freshly redissolved in sterile deionized water by sonication at 40 kHz for 15 min with on–off cycles of 15 s each. Cell viability was determined by XTT-based cytotoxicity assay following the manufacturer’s instructions.

### Total RNA Extraction and Real-Time PCR Analysis

Total RNA was extracted using the RNeasy Mini kit (Qiagen) and transcribed to cDNA using the High-Capacity cDNA Reverse Transcription Kit (Applied Biosystems) following the manufacturer’s protocol. Expression of hBD2 (*DEFB4A*, forward: 5′-CCC TTT CTG AAT CCG C-3′, reverse: 5′-GAG GGT TTG TAT CTC CT-3′), RNase7 (*RNASE7*, forward: 5′-GGA GTC ACA GCA CGA AGA CCA-3′, reverse: 5′-CAT GGC TGA GTT GCA TGC TTG A-3′), psoriasin (*S100A7*, forward: 5′-GGC TAT GTC TCCCAG CAA-3′, reverse: 5′-CAC CAG ACG TGA TGA CAA-3′) was analyzed using SYBR Green reagent in Rotor-Gene PCR cycler (Corbett Life Science). Human beta-actin (*ACTB*, forward: 5′-AAG AGA GGC ATC CTC ACC CT-3′, reverse: 5′-TAC ATC GCT GGG GTG TTG-3′) was used as the housekeeping gene to calculate relative gene expression.

### Immunofluorescence Microscopy

HaCaT were treated with Ag@ZnO for 24 h, fixed with 4% paraformaldehyde, and stained with anti-hBD2 antibody (Santa Cruz Biotechnology), anti-RNase7 antibody (Novus Biologicals) at 1:100 dilution, followed by respective Alexa Fluor 488 secondary antibody (Life Technologies) at 1:1000 dilution and counterstained with 4′,6-diamidino-2-phenylindole (DAPI; Life Technologies). Confocal imaging was performed using the 63× oil immersion objective of an LSM 700 microscope (Carl Zeiss).

For quantifying wound closure, a straight line scratch was made on a fully confluent HaCaT layer and incubated ± Ag@ZnO for 72 h. Cells were fixed and imaged using the 20× objective of an LSM 700 microscope. For Ki67 and vinculin staining, HaCaT cells were incubated ± Ag@ZnO for 24 h, stained with 1:200 dilution of primary (AbCam) and 1:1000 dilution of Alexa Fluor secondary antibodies (Life Technologies), and counter-stained with DAPI. Imaging was performed with the 40× water-immersion objective in the 3 × 2 tile mode of an LSM 700 microscope. All images were processed using LSM image examiner (Carl Zeiss) or ImageJ (NIH).

### Isolation of Bacteria from Wounds

*S*. *aureus* type strain ATCC 29213 and clinical *S*. *aureus* isolates from wounds of 50 individuals at the Karolinska University Hospital or associated outpatient clinics were obtained and cultured overnight on blood agar at 37 °C for the assays.

### Antibacterial Assay

The antibacterial efficacy of Ag@ZnO was determined using a broth microdilution method. Ag@ZnO, at indicated concentration, was diluted in tryptic soy broth with 0.25% glucose and inoculated with 5 × 10^5^ CFU/mL of *S*. *aureus*. Bacteria were incubated for 24 h in 37 °C, serially diluted, and plated for viable count.

### Intracellular Bacterial Killing Assay

Cells were infected with *S*. *aureus* type strain at MOI 30 for 30 min. Extracellular bacteria were eliminated by 1 h gentamycin (100 μg/mL) treatment. Cells were treated with Ag@ZnO, lysed, and plated. Bacterial survival was calculated in comparison to initial invaded bacteria at 0 h and represented as fold change normalized to untreated cells.

### Conditioned Media Bacterial Killing Assay

Post stimulation of cells with Ag@ZnO, the residual cells and nanoparticles were removed by centrifugation of conditioned media at 12 000*g* for 5 min. Fifty microliters from 10^4^ CFU/mL of ATCC 29213 was added to 150 μL of the conditioned media, vortexed, incubated for 6 h, and plated.

### Lysosomal Targeting Assays

HaCaT cells were infected with *S*. *aureus* type strain prestained with bacterial viability stain SYTO-9 (Life Technologies) following the manufacturer’s instructions. After 1.5 h of incubation with 100 μg/mL of Ag@ZnO, lysosomes were stained with LysoTracker Red (1 μM, 20 min, Life Technologies), fixed, and counterstained with DAPI.

### Statistical Analysis

For all assays, three independent experiments were performed in either duplicates, triplicates, or quadruplets. Comparison among multiple groups was done by one-way ANOVA with Dunnett’s multiple comparison test. Comparison between two groups was performed with Wilcoxon signed-rank test unless otherwise stated. All statistical tests were performed in Graph Pad Prism version 5, considering *P* < 0.05 as statistically significant.
